# Characterization of genome-wide H3K27ac profiles reveals a distinct PM_2.5_-associated histone modification signature

**DOI:** 10.1186/s12940-015-0052-5

**Published:** 2015-08-15

**Authors:** Cong Liu, Junhui Xu, Yahong Chen, Xinbiao Guo, Yinan Zheng, Qianfei Wang, Yiyong Chen, Yang Ni, Yidan Zhu, Brian Thomas Joyce, Andrea Baccarelli, Furong Deng, Wei Zhang, Lifang Hou

**Affiliations:** Department of Bioengineering, University of Illinois at Chicago, Chicago, IL USA; Department of Occupational and Environmental Health Sciences, School of Public Health, Peking University, Beijing, 100191 China; Respiratory Department, Peking University Third Hospital, Beijing, China; Institute for Public Health and Medicine, Northwestern University Feinberg School of Medicine, Chicago, IL USA; Department of Preventive Medicine, Northwestern University Feinberg School of Medicine, Chicago, IL USA; Division of Epidemiology/Biostatistics, School of Public Health, University of Illinois at Chicago, Chicago, IL USA; Department of Environmental Health, Harvard T.H. Chan School of Public Health, Boston, MA USA; The Robert H. Lurie Comprehensive Cancer Center, Northwestern University Feinberg School of Medicine, 680 N. Lake Shore Dr., Suite 1400, Chicago, IL 60611 USA; Key Laboratory of Genomic and Precision Medicine, Beijing Institute of Genomics, Chinese Academy of Sciences, Beijing, 100101 China

**Keywords:** Histone modification, H3K27ac, Particulate matter, Epigenetics, Environmental health, Gene regulation

## Abstract

**Background:**

Current studies of environmental health suggest a link between air pollution components, such as particulate matter (PM), and various diseases. However, the specific genes and regulatory mechanisms implicated in PM-induced diseases remain largely unknown. Epigenetic systems such as covalent modification of histones in chromatin may mediate environmental factors in gene regulation. Investigating the relationships between PM exposure and histone modification status may help understand the mechanisms underlying environment-associated health conditions.

**Methods:**

In this study, we obtained genome-wide profiles of H3K27ac (histone 3 lysine 27 acetylation), known to be an active gene regulatory histone modification marker, in blood samples collected from four Chinese individuals exposed to high or low PM_2.5_ (particles with diameters up to 2.5 μm).

**Results:**

The genome-wide chromatin immunoprecipitation sequencing (ChIP-Seq) data indicated a comprehensive differential H3K27ac landscape across the individual genomes, which was associated with high PM_2.5_. Moreover, a substantial number of these PM_2.5_-associated differential H3K27ac markers were in genes involved in immune cell activation, potentially linking these epigenetic changes with air pollution-induced immune and inflammatory responses.

**Conclusions:**

Our study provides the first genome-wide characterization of H3K27ac profiles in individuals subjected to different exposure levels of PM_2.5_. Future systematic investigations of the relationships between air pollutants and histone modifications in large population samples are warranted to elucidate the contributions of histone modifications to environment-associated diseases.

**Electronic supplementary material:**

The online version of this article (doi:10.1186/s12940-015-0052-5) contains supplementary material, which is available to authorized users.

## Background

Air pollutants have been demonstrated to exert significant adverse health effects in populations around the world. Particulate matter (PM), which represents a mixture of solid particles and liquid droplets found in the air, in particular has been associated with increased morbidity and mortality from various diseases [1–5]. Gene dysregulation plays a fundamental role in disease pathogenesis and development. Thus investigating gene dysregulation mechanisms related to PM exposure may enhance our knowledge of air pollution-related health conditions, providing important information for disease prevention, diagnosis and treatment.

Covalent histone modifications, such as methylation and acetylation of certain amino acid residues in chromatin histones, have been shown to play an essential role in gene regulatory function by modulating chromatin structures. For example, the Encyclopedia of DNA Elements (ENCODE) Project [6] has systematically identified histone modification markers with distinct gene regulatory roles in the human genome (e.g., activation by H3K4me3 - histone 3 lysine 4 tri-methylation; repression by H3K9me3 - histone 3 lysine 9 tri-methylation) [7].

Furthermore, epigenetic changes including histone modifications are increasingly being linked with gene dysregulation and cellular responses induced by air pollutants, including ambient PM [8]. PM exposure was found to promote the release of inflammatory cytokines, which is further enhanced by co-treatment with a histone deacetylase inhibitor [9], indicating that differential histone acetylation could be involved in PM-mediated pro-inflammatory responses. Some reports also indicated that PM-containing environmental contaminants (e.g., nickel, chromium) contribute to dysregulated histone acetylation [10, 11]. Since there are different types of histone modifications, the ultimate effects of histone modifications related to air pollutant exposure remain unclear, and likely depend upon the exact composition of the PM. In addition, genome-wide histone modification patterns induced by air pollution has yet to be characterized. Therefore, in this study, our objective was to characterize the modification patterns of H3K27ac (histone 3 lysine 27 acetylation) associated with PM_2.5_ (particles with diameters up to 2.5 μm). H3K27ac has been identified as an active regulatory histone modification marker with a putative role in separating active enhancers from their poised counterparts [12]. Specifically, we used the unbiased, genome-wide Chromatin Immunoprecipitation Sequencing (ChIP-Seq) to profile H3K27ac markers across the genomes of individuals with varying PM_2.5_ exposure levels. Individual histone modification profiles were compared between the exposure groups to provide an overall landscape of differential H3K27ac markers associated with high PM_2.5_ exposure. Genes that may be regulated by these PM_2.5_-associated histone markers were then evaluated for their potential functions and impacts on human complex diseases/traits, by taking advantage of publicly available functional annotation databases and genome-wide association study (GWAS) results.

## Methods

### Study subjects, sample preparation, and ChIP-Seq assay

Four healthy subjects were assigned into low or high exposure groups according to measurements of outdoor PM_2.5_ levels (Table 1). All study participants are Han Chinese who worked and lived in the Beijing metropolitan area. This study was approved and exempted by the Institutional Review Board of each collaborating institution with written informed consent obtained from all subjects.

**Table 1:** Study subjects

Subject ID	Exposure group	Outdoor PM_2.5_ (μg/m^3^)	Indoor PM_2.5_ (μg/m^3^)
1	low	7	15
2	low	9	17
3	high	22	105
4	high	52	131

Nuclei from polymorphonuclear leukocytes of subjects were extracted using PolymorphPrep™ (Axis-Shield, Dundee, UK). These extracted nuclei were then lysed and sonicated to produce sheared chromatin 200-600 bp long. The quality of the sheared chromatin, and sonication efficiency, were checked according to standard molecular biology protocols. The ChIP-Seq assay was then used to profile the modification levels of H3K27ac in each individual’s genomes. Briefly, the final soluble chromatin was prebound with an H3K27ac antibody (Abcam, Cambridge, UK; #ab4729). Whole-cell extract (WCE) samples untreated with the antibody (i.e., the input samples) were retained as controls. The immunoprecipitated chromatin was washed, purified and eluted. The crosslinks were then reversed. The purified DNA were prepared for sequencing using the ChIP-Seq kit according to the manufacturer’s protocol (Illumina, Inc., San Diego, CA). Sequencing was performed using the Illumine HiSeq2000 platform (Illumina, Inc., San Diego, CA). The raw ChIP-Seq data have been deposited into the NCBI Sequence Read Archive (Accession Number: SRP057970).

### ChIP-Seq data processing and characterization

Histone modification peaks were identified from the raw ChIP-Seq data using the next-generation sequencing analysis tools provided in the Galaxy Project [13]. Low quality reads as more than 10 % of bases with quality scores less than 20 were filtered. The cleaned 101 bp single-end sequencing reads were mapped to the human genome reference (hg19) using Bowtie2 [14]. Only non-redundant and uniquely mapped reads were retained to correct for sequencing bias. To define the H3K27ac enriched genomic regions (peaks), the model-based algorithm MACS [15] was used to compare the ChIP-Seq signal to its corresponding input sample. Peaks with overlaps in different individuals were merged into a broad peak domain using BEDTools [16]. Differential H3K27ac loci were defined as broad peaks detected in one group that were missing in another group.

To characterize the general distribution of H3K27ac profiles in these individuals, aggregate H3K27ac profiles were generated using an in-house script. The human RefSeq [17] transcription start site (TSS) annotations and the ENCODE ChromHMM-detected enhancers derived for the lymphoblastoid cell line GM12878 [18] were downloaded from the UCSC Genome Browser (https://genome.ucsc.edu/). A window of +/− 10 kb from each TSS or center of each enhancer was split into 400 bins (50 bp/bin). For each bin, the normalized reads density was calculated as a log ratio of the average reads density for the ChIP sample to its corresponding input sample, thus generating a genome-wide H3K27ac intensity profile for either promoters or enhancers.

### Functional annotation analyses

Functions of differential H3K27ac loci were predicted by analyzing the annotations of nearby genes using the Genomic Regions Enrichment of Annotations Tool (GREAT) [19]. In particular, each gene was assigned a basal regulatory domain from 5 kb upstream to 1 kb downstream of the TSS. The gene regulatory domain was extended in both directions to the next nearest gene's basal domain but no more than 100 kb in one direction. Each differential H3K27ac locus was associated with all genes whose regulatory domain it overlapped. Significantly enriched Gene Ontology (GO) [20] biological processes and PANTHER pathways [21] were identified under 5 % false discovery rate (FDR) using a hypergeometric test. To evaluate PM_2.5_-associated epigenetic effects on human complex diseases/traits, differential H3K27ac loci were overlapped with trait-associated genetic variants, i.e., single nucleotide polymorphisms (SNPs) from the NHGRI GWAS Catalogue [22], which contains over 1700 curated publications of more than 12000 SNPs (Catalog Data: February 20, 2015). Significant GWAS SNP-trait associations were limited to those with nominal p-values smaller than 1.0 × 10^−5^, as reported by the GWAS Catalogue.

## Results

**Fig. 1:**
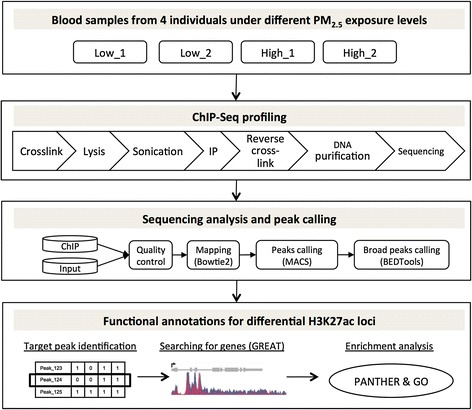
Overview of the study design and analysis workflow. Nuclei were extracted from blood samples of four individuals under low or high exposure of outdoor PM_2.5_. Standard protocol was used for ChIP-Seq experiments. Bioinformatics software (in brackets) and in-house scripts were used to analyze the sequencing data. The functions of differential H3K27ac loci were evaluated using public databases. ChIP: chromatin immunoprecipitation; IP: immunoprecipitation; GO: gene ontology

Figure 1 shows the general workflow of this study. Briefly, four individuals subjected to different PM_2.5_ exposure levels were profiled for genome-wide H3K27ac profiles using ChIP-Seq. A standard ChIP-Seq data analysis pipeline was performed to identify differential histone modification loci between the two exposure groups.

**Fig. 2:**
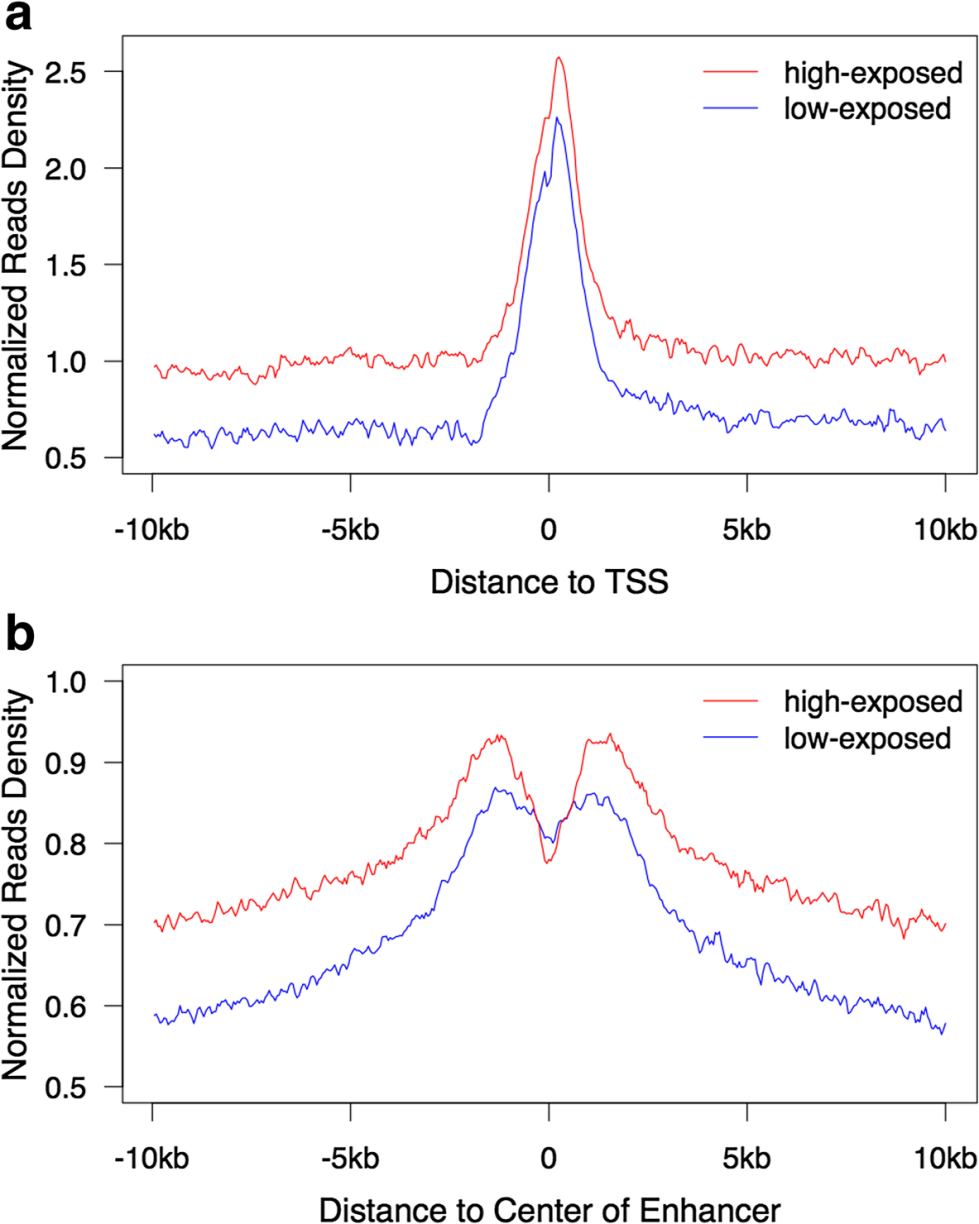
Global profiles of H3K27ac overlapped with promoters and enhancers. Global H3K27ac signal densities normalized by input were determined in a 20 kb-window surrounding **a** the center of ENCODE-detected enhancers; and **b** the TSS of human RefSeq genes. The red curve represents the average H3K27ac signal density in the high-exposed group and the blue curve represents the average density in the low-exposed group. Both promoters and enhancers show global elevated H3K27ac modification levels in the individuals exposed to higher PM_2.5_ (red). TSS: transcription start site

For each individual, both ChIP and input samples were sequenced. After conventional quality control, around 2–9 million unique reads were mapped to the reference genome (hg19) in the ChIP samples, in contrast to the 4–12 million reads in the input samples (Additional file 1: Table S1). In total, 7000 ~ 54000 peaks were called using a stringent peak detection threshold p-value of *p* < 10^−5^ (Additional file 2: Table S2). Among differentially modified H3K27ac loci, 1080 loci were induced in the group with high PM_2.5_ exposure, and 158 loci were suppressed (Additional file 3: Table S3). In general, individuals with higher PM_2.5_ tended to have a higher number of peaks. In addition, a similar global pattern was observed from aggregation plots of H3K27ac on promoter and enhancer regions (Fig. 2). H3K27ac peaks were clearly overlapped in promoter and enhancer regions, consistent with the putative role of H3K27ac as a promoter and enhancer marker. Both the TSS and enhancer peaks were higher in the individuals with high PM_2.5_ exposure compared to low-exposed individuals. These findings could indicate global enhancement of gene expression due to the exposure to PM_2.5_ pollutants.

**Fig. 3:**
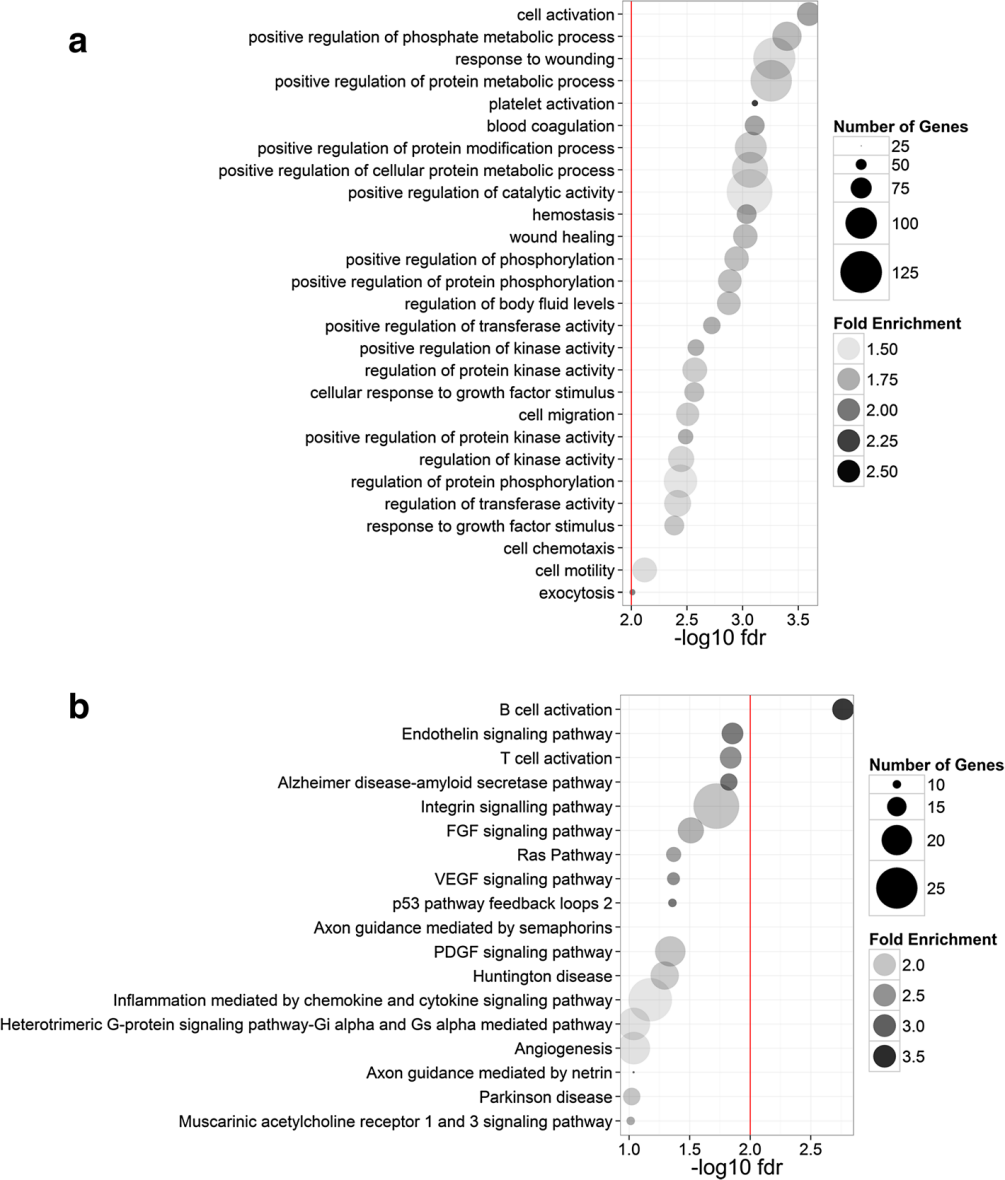
Enriched functional annotations among differential H3K27ac loci. Proximal gene enrichments for differential H3K27ac loci were analyzed using **a** GO biological processes; and **b** PANTHER pathways. Circle size is proportional to the number of identified genes. Circle transparency is proportional to fold enrichment relative to the human genome. Functional annotations were ordered by FDR derived from hypergeometric test. Red vertical lines indicate the cutoff at 5 % FDR. GO: gene ontology; FDR: false discovery rate

Gene set enrichment analysis was performed to evaluate the genes annotated by the identified differential H3K27ac loci using the GREAT tools. Additional file 4: Table S4 shows significantly enriched GO biological processes and PANTHER pathways. We found that most of the associated genes were involved in the activation of cellular responses to wounding and stimulus, suggesting an enhancer-mediated cell activation mechanism in response to higher PM_2.5_ exposure. We also found differential H3K27ac loci were most significantly enriched in pathways related to immune response, including T-cell and B-cell activation (Fig. 3). Interestingly, we found that a pathway related to Alzheimer’s disease was enriched in our results.

A previous study showed that complex trait-associated variants were enriched in specific histone marks [23]. We also found 11 complex trait-associated genetic variants overlapping with our identified PM_2.5_-associated epigenetic signature (Table 2). Diseases such as Alzheimer’s disease and inflammatory bowel disease (IBD) were found to have overlapping GWAS-identified loci with our PM_2.5_-associated H3K27ac markers.

**Table 2:** Overlap of complex trait-associated genetic variants and the identified PM_2.5_-asociated H3K27ac loci

Complex trait	SNP ID	Region	Reported gene	Context	PubMed reference
Inflammatory bowel disease	rs12654812	5q35.3	DOK3	intron	23128233
Renal function-related traits	rs12654812	5q35.3	RGS14	intron	22797727
Suicide attempts in depression or bipolar disorder	rs17173608	7q36.1	RARRES2	intron	24964207
IgG glycosylation	rs11994937	8q12.1	-	intron	23382691
Fibrinogen	rs7464572	8q24.3	PLEC1	intron	23969696
Metabolic traits	rs6558295	8q24.3	OPLAH	intron	21886157
Adverse response to chemotherapy (neutropenia/leucopenia) (paclitaxel + carboplatin)	rs10785877	9q34.2	RXRA	-	23648065
Alzheimer’s disease	rs3764650	19p13.3	ABCA7	intron	21460840
	rs115550680	19p13.3	ABCA7, HMHA1, GRIN3B	intron	23571587
Red blood cell traits	rs737092	20q13.31	RBM38	-	23222517
Cognitive decline (age-related)	rs9980664	21q22.11	Intergenic	-	24468470

## Discussion

Accumulating evidence has demonstrated that PM is able to induce systemic inflammatory responses by altering the expression of specific genes [24–28]. For example, Ovrevik, et al. [26] found that particulate air pollution up-regulated inflammation-related chemokines and cytokines in a bronchial epithelial cell line. In a paired sampling study design, Wang, et al. [25] showed that levels of systemic inflammatory responses were significantly increased in the peripheral blood of a population exposed to PM_2.5_. Our observation of PM_2.5_-associated epigenetic change is consistent with previous studies. Specifically, various biological processes related to inflammatory or immune cell activation and inflammation pathways such as CXC-chemokine receptors appeared to be activated via altered acetylation levels of H3K27 occurring after exposure to PM_2.5_.

PM_2.5_ is also notoriously implicated in blood coagulation. Previous studies [29, 30] demonstrated that inhalation of some PM components may be responsible for altering red cell adhesiveness, inducing endothelial dysfunction, and increasing blood coagulation, all of which offers biological mechanisms for the observed cardiovascular effects of particulate air pollution exposure. Our study found that increased exposures to PM_2.5_ might cause increased acetylation levels of H3K27ac markers for specific genes involved in platelet activation, blood coagulation and hemostasis. Certain pathways are likely to be activated via epigenetic regulation, thus leading to increased coagulation of red cells, leading to various cardiovascular diseases in turn.

Air pollution has been considered a risk factor for both complex neurodegenerative diseases such as Alzheimer's disease and Parkinson's disease, and monogenic neurological disorders such as Huntington's disease [31, 4, 32]. Our observation that there is an interaction between PM_2.5_ and epigenetic regulation offers a possible explanation for these nervous system diseases. Certain differential H3K27ac loci are potential regulators for genes involved in the Alzheimer’s disease-amyloid secretase pathway, Huntington disease and Parkinson disease. Two Alzheimer’s disease-associated genetic variants (rs3764650, rs115550680) [33, 34] were found to fall into the differential H3K27ac loci in our study. Another variant (rs17173608) associated with depression and bipolar disorder [35] was also found to overlap with a PM_2.5_-associated enhancer region. Our results suggest that PM_2.5_ may play an important role in deregulating nervous system functions via its ability to alter the acetylation levels of related enhancers.

By linking differentially modified H3K27ac loci to complex-trait loci identified by GWAS studies [22], we found that PM_2.5_-associated epigenetic changes may help improve our understanding of human complex diseases/traits. For example, ambient air pollution has been reported to correlate with hospitalizations for IBD [36], while our finding suggested that a previously identified inflammatory bowel disease-associated variant (rs12654812) overlapped with a PM_2.5_-associated H3K27ac locus, thus air pollution-associated IBD is likely mediated through this histone modification marker.

## Conclusions

Our study constitutes the first genome-wide characterization of H3K27ac profiles in individuals who are subjected to different exposure levels of PM_2.5_. Our findings reveal a global elevation of the enhancer-associated H3K27ac markers in individuals exposed to relatively high levels of PM_2.5_. Furthermore, certain immune response and inflammation-related genes are likely mediated via H3K27ac makers under PM_2.5_ exposure. We recognize that this study had a number of limitations. Due to the limited sample size, it may not be plausible to draw firm conclusions from these data yet. In addition, epigenetic markers may be affected by possible confounding factors, such as age, gender, and race. Statistically, potential false positives from conducting multiple tests are a concern as with any high-throughput technology. However, as the first genome-wide H3K27ac landscape in people exposed to high levels of PM_2.5,_ our investigation suggests a trend that increasing exposure to PM_2.5_ may enhance global gene activity. Changes in certain H3K27ac modification loci may affect local gene expression, which in turn could lead to a variety of diseases. Large-scale studies in the future are warranted to comprehensively evaluate and validate the genes and pathways influenced by PM_2.5_ through H3K27ac and other histone modifications.

## Additional files

Additional file 1: Table S1. Summary of sequencing and mapping results. (DOCX 20 kb) Additional file 2: Table S2. Summary of peak calling results. (DOCX 14 kb) Additional file 3: Table S3. Complete list of differentially modified H3K27ac loci. (XLSX 69 kb) Additional file 4: Table S4. Complete list of functional annotations. (XLSX 16 kb)

## Abbreviations

ChIP: Chromatin immunoprecipitation
ENCODE: Encyclopedia of DNA elements
GO: Gene ontology
GREAT: Genomic regions enrichment of annotations tool
GWAS: Genome-wide association study
H3K27ac: Histone 3 lysine 27 acetylation
H3K4me3: Histone 3 lysine 4 tri-methylation
H3K9me3: Histone 3 lysine 9 tri-methylation
IBD: Inflammatory bowel disease
PM: Particulate matter
PM_2.5_: Particles with diameters up to 2.5 μm
SNP: Single nucleotide polymorphism
TSS: Transcription start site
WCE: Whole-cell extract
