# Supplementary material for: Characterization of genome-wide H3K27ac profiles reveals a distinct PM2.5-associated histone modification signature
Source: Environ Health. 2015 Aug 15;14:65. doi: 10.1186/s12940-015-0052-5 (PMC4537530; doi:10.1186/s12940-015-0052-5)
Supplement: Additional file 1: Table S1. — Summary of sequencing and mapping results. (DOCX 20 kb) [file 12940_2015_52_MOESM1_ESM.docx]

**Table S1 Summary of sequencing and mapping results**

| **Subject ID** | **Exposure group** | **Experiment** | **Raw^a^** | **Cleaned^b^** | **Unique^c^** | **Non-redundant^d^** |
| --- | --- | --- | --- | --- | --- | --- |
| 1 | low | ChIP | 23,876,993 | 15,521,297 | 3,516,305 | 3,132,911 |
|  |  | input | 10,427,040 | 8,968,210 | 5,972,122 | 5,791,107 |
| 2 | low | ChIP | 30,485,788 | 20,847,560 | 10,044,142 | 8,969,292 |
|  |  | input | 10,055,696 | 7,549,958 | 4,415,208 | 4,312,592 |
| 3 | high | ChIP | 24,223,516 | 8,911,903 | 5,550,599 | 4,537,953 |
|  |  | input | 13,426,622 | 10,563,234 | 7,714,193 | 6,807,520 |
| 4 | high | ChIP | 15,814,022 | 5,038,505 | 2,668,567 | 2,101,112 |
|  |  | input | 23,122,482 | 18,184,920 | 13,223,276 | 12,435,939 |

^a^ Count of raw DNA reads obtained from sequencing;

^b^ Count of cleaned DNA reads after filter reads with low quality;

^c^ Count of DNA reads mapped uniquely to one genomic coordinate;

^d^ Count of DNA reads after removing PCR duplicates.
