# Supplementary material for: Characterization of genome-wide H3K27ac profiles reveals a distinct PM2.5-associated histone modification signature
Source: Environ Health. 2015 Aug 15;14:65. doi: 10.1186/s12940-015-0052-5 (PMC4537530; doi:10.1186/s12940-015-0052-5)
Supplement: Additional file 2: Table S2. — Summary of peak calling results. (DOCX 14 kb) [file 12940_2015_52_MOESM2_ESM.docx]

**Table S2 Summary of peak calling results**

| **Subject ID** | **Exposure group** | **Peaks (p<10^-5^)** | **Broad peaks (p<10^-5^)** |
| --- | --- | --- | --- |
| 1 | low | 7,618 | 6,958 |
| 2 | low | 11,115 | 10,078 |
| 3 | high | 54,483 | 53,480 |
| 4 | high | 7,659 | 7,344 |
